# Saikosaponin A and Saikosaponin C Reduce TNF-α-Induced TSLP Expression through Inhibition of MAPK-Mediated EGR1 Expression in HaCaT Keratinocytes

**DOI:** 10.3390/ijms23094857

**Published:** 2022-04-27

**Authors:** Sung Shin Ahn, Young Han Lee, Hyunjin Yeo, Euitaek Jung, Yoongho Lim, Soon Young Shin

**Affiliations:** 1 Department of Biological Sciences, Sanghuh College of Lifesciences, Konkuk University, Seoul 05029, Korea; wendy713@konkuk.ac.kr (S.S.A.); yhlee58@konkuk.ac.kr (Y.H.L.); jini1606@konkuk.ac.kr (H.Y.); mylife4sci@konkuk.ac.kr (E.J.); 2 Division of Bioscience and Biotechnology, BMIC, Konkuk University, Seoul 05029, Korea; yoongho@konkuk.ac.kr

**Keywords:** atopic dermatitis, saikosaponin, early growth response 1, mitogen-activated protein kinase, thymic stromal lymphopoietin, tumor necrosis factor-α

## Abstract

Atopic dermatitis (AD) is one of the most common chronic inflammatory skin diseases worldwide, characterized by intense pruritus and eczematous lesions. Aberrant expression of thymic stromal lymphopoietin (TSLP) in keratinocytes is associated with the pathogenesis of AD and is considered a therapeutic target for the treatment of this disease. Saikosaponin A (SSA) and saikosaponin C (SSC), identified from *Radix Bupleuri*, exert anti-inflammatory effects. However, the topical effects of SSA and SSC on chronic inflammatory skin diseases are unclear. In this study, we investigated the effects of SSA and SSC on TSLP suppression in an AD-like inflammatory environment. We observed that SSA and SSC suppressed tumor necrosis factor-α-induced TSLP expression by downregulating the expression of the transcription factor early growth response 1 (EGR1) via inhibition of the extracellular signal-regulated kinase 1/2, c-Jun N-terminal kinase 1/2, and p38 mitogen-activated protein kinase pathways. We also confirmed that topical application of SSA or SSC reduced AD-like skin lesions in BALB/c mice challenged with 2,4-dinitrochlorobenzene. Our findings suggest that suppression of EGR1-regulated TSLP expression in keratinocytes might be attributable to the anti-inflammatory effects of SSA and SSC in AD-like skin lesions.

## 1. Introduction

Atopic dermatitis (AD) is one of the most common chronic inflammatory skin diseases worldwide. It is characterized by intense pruritus and eczematous lesions, which considerably affect the quality of life of patients [1,2]. However, the exact causes of AD are not fully understood [1,2]. A growing body of evidence suggests that the onset of AD is related to a complex interplay among genetic factors, lifestyle factors, host environmental risk factors, skin inflammation, and impairment of epidermal barrier function [3]. The pathogenesis of AD is primarily caused by multiple inflammatory cytokines released from various inflammatory cells, including CD4^+^ T-helper (Th) lymphocytes, mast cells, monocytes, and neutrophils [4]. Analyses of biopsies from patients with AD demonstrated an increased number of Th2 lymphocytes expressing interleukin (IL)-4 and IL-13 [5]. Furthermore, in animal models of AD, eczematous skin lesions do not occur without Th1 and Th2 lymphocytes [5], suggesting that CD4^+^ Th lymphocyte-derived cytokines enhance skin inflammatory responses and the progression of AD skin lesions [6].

Thymic stromal lymphopoietin (TSLP) is an IL-7-like cytokine expressed mainly by stromal and epithelial cells in the skin, lung, and intestine [7,8]. TSLP acts on dendritic cells to induce Th cell development, promoting Th2 cytokine-mediated inflammation in inflammatory diseases, such as AD, allergic rhinitis, and asthma [9]. In acute and chronic AD skin lesions, TSLP is increasingly released from keratinocytes and triggers dendritic cell-mediated allergic inflammation and initiation of Th2 immune responses [8,10,11,12]. Furthermore, TSLP directly binds to sensory neurons and induces itch sensitization [13]. Since upregulation of keratinocyte-derived TSLP expression is involved in Th2 priming and progression of skin inflammation, aberrant expression of TSLP has been considered a crucial biomarker in the pathogenesis of AD [8,14,15]. Therefore, to develop better therapeutic strategies for AD, it is necessary to understand the regulatory mechanisms of TSLP expression and inhibit its upregulation in the inflammatory skin environment.

Natural products are a rich source of novel drug candidates with fewer side effects than those of many synthetic drugs [16,17]. *Radix Bupleuri*, the dried root of *Bupleurum* species, has been widely used as an oriental folk medicine [18]. Its extract exerts various pharmacological effects, including antiviral, anticancer, hepatoprotective, immunomodulatory, and anti-inflammatory activities [18,19]. Saikosaponins are bioactive oleanane-type triterpenoid saponins isolated from *Radix Bupleuri* [19]. To date, more than 100 different saikosaponins have been identified from *Radix Bupleuri*. However, there is little information regarding the anti-inflammatory activity of saikosaponins against chronic skin inflammatory diseases, such as AD. Of these, saikosaponin A (SSA, CAS 20736-09-8) and saikosaponin C (SSC, CAS 20736-08-7) exert anti-inflammatory effects [18,19,20,21,22]. However, the topical effects of SSA and SSC on chronic skin inflammatory diseases such as AD are unclear.

In this study, we investigated the biological activities of SSA and SSC in the regulation of TSLP expression in TNF-α-stimulated HaCaT keratinocytes. We also evaluated the potential of SSA and SSC for topical application in allergic skin inflammatory diseases using a 2,4-dinitrochlorobenzene (DNCB)-challenged BALB/c mouse model. 

## 2. Results

### 2.1. Effects of SSA and SSC on Cytotoxicity in HaCaT Keratinocytes

**Figure 1:**
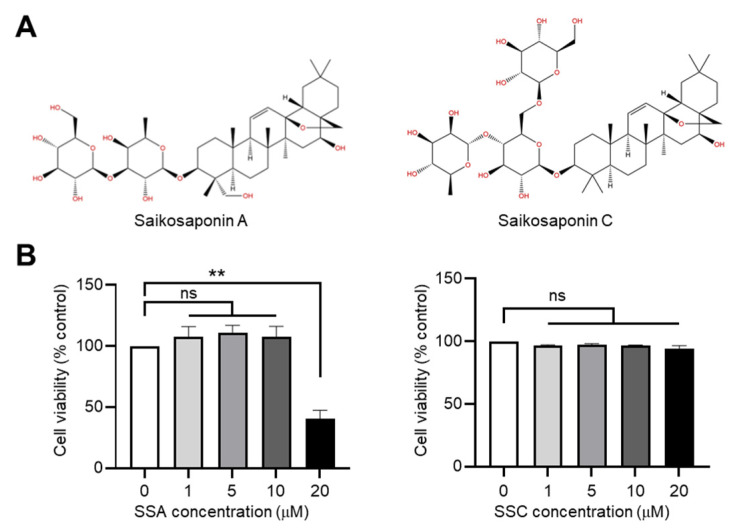
Cytotoxic effects of saikosaponin A (SSA) and C (SSC) in HaCaT cells. (**A**) Chemical structures of SSA and SSC. (**B**) HaCaT cells were treated with different concentrations (0, 1, 5, 10, 20 μM) of SSA or SSC for 24 h. Cell viability was measured by performing the cell counting kit-8 assay. Data are expressed as mean ± SD (*n* = 3). ns, not significant; ** *p* < 0.01 by Sidak’s multiple comparisons test.

We first determined the effects of SSA and SSC (Figure 1A) on cytotoxicity in HaCaT cells by performing the cell counting kit-8 (CCK-8) assay based on water-soluble formazan. We observed that SSA and SSC were not cytotoxic at concentrations up to 10 µM and 20 µM, respectively, in HaCaT keratinocytes (Figure 1B). Therefore, in the following experiments, we used concentrations that were not cytotoxic; 0.5 or 1 µM for SSA and 10 or 20 µM for SSC. 

### 2.2. SSA and SSC Abrogate TNF-α-Induced TSLP Expression at the mRNA and Protein Levels

Keratinocyte-derived TSLP is a master regulator of Th2 inflammatory responses in AD. Thus, upregulated TSLP expression is considered a hallmark of AD pathogenesis [14,15]. TNF-α is a pro-inflammatory cytokine that promotes the production of multiple inflammatory cytokines and chemokines [23]. TNF-α was highly expressed in a mouse model of DNCB-induced contact allergy [24], and it induces TSLP expression in keratinocytes [25].

**Figure 2:**
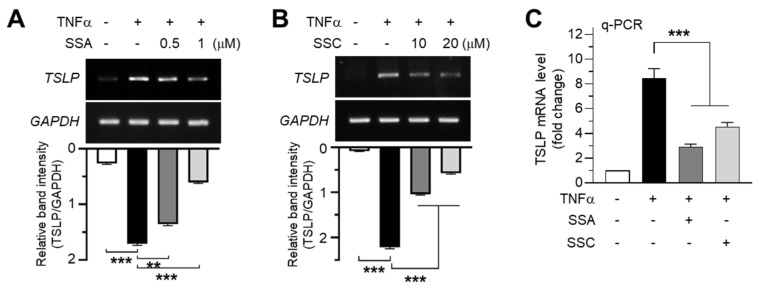
Inhibitory effects of SSA and SSC on TNF-α-induced TSLP expression in HaCaT cells. (**A**,**B**) HaCaT cells were pretreated with 0.5 or 1 μM SSA (**A**) and 10 or 20 μM SSC (**B**) for 30 min before treatment with 10 ng/mL of TNF-α. After 12 h, TSLP mRNA levels were measured using reverse transcription-PCR. The band intensities corresponding to TSLP levels were normalized to those of GAPDH levels using ImageJ software. (**C**) HaCaT cells were treated with 10 ng/mL of TNF-α for 12 h in the absence or presence of 1 μM SSA or 20 μM SSC, and quantitative real-time PCR was performed. Data are expressed as means ± SD (*n* = 3). ** *p* < 0.01, *** *p* < 0.001 by Dunnett’s multiple comparisons test.

To investigate whether *TSLP* expression is altered upon exposure to SSA or SSC, we treated HaCaT keratinocytes with TNF-α and analyzed *TSLP* mRNA expression in these cells. Consistent with a previous report [25], the TNF-α-induced increase in *TSLP* mRNA levels was reduced by pretreatment with SSA (Figure 2A) or SSC (Figure 2B) in a dose-dependent manner as revealed by reverse transcription-PCR (RT-PCR). Quantitative real-time PCR (qPCR) analysis showed that TNF-α increased *TSLP* mRNA levels by 8.45-fold compared to those of the control (Figure 2C), which was significantly (*p* < 0.001) decreased by pretreatment with 1 µM SSA or 10 µM SSC. 

**Figure 3:**
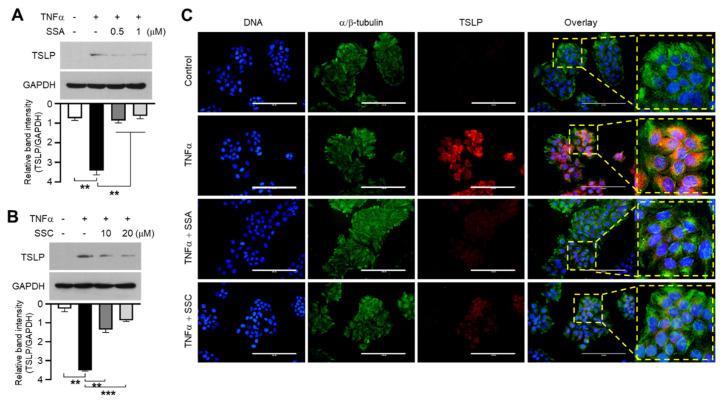
SSA and SSC decrease TNF-α-induced increase in TSLP protein levels in HaCaT cells. (**A**,**B**) HaCaT cells were pretreated with 0.5 or 1 μM SSA (**A**) and 10 or 20 μM SSC (**B**) for 30 min before treatment with 10 ng/mL of TNF-α. After 24 h, TSLP protein levels were measured by immunoblot analysis. The band intensities corresponding to TSLP levels were normalized to those of GAPDH levels using ImageJ software. The quantitation data are presented as means ± SD (*n* = 3). ** *p* < 0.01 *** *p* < 0.001 by Sidak’s multiple comparisons test. (**C**) HaCaT cells, cultured on coverslips, were treated with TNF-α (10 ng/mL) for 24 h in the absence or presence of SSA (1 μM) or SSC (20 μM), and immunofluorescence staining was performed using anti-TSLP (red) and anti-α/β-tubulin (green) antibodies. Nuclei were counterstained using 1 µg/mL Hoechst 33258 (blue). Scale bars, 200 μm.

To determine whether SSA and SSC reduced TNF-α-induced increase in TSLP protein levels, we performed immunoblot analysis. Consistent with their mRNA levels, TNF-α-induced enhanced protein expression of TSLP was markedly decreased after pretreatment with SSA (Figure 3A) or SSC (Figure 3B). Fluorescence immunocytochemical staining confirmed that TNF-α-induced cytoplasmic localization of TSLP was markedly decreased in the presence of SSA or SSC (Figure 3C). These data suggest that SSA and SSC can reduce TNF-α-induced TSLP expression at both the mRNA and protein levels in HaCaT keratinocytes. 

### 2.3. SSA and SSC Abrogate TNF-α-Induced TSLP Promoter Activity

**Figure 4:**
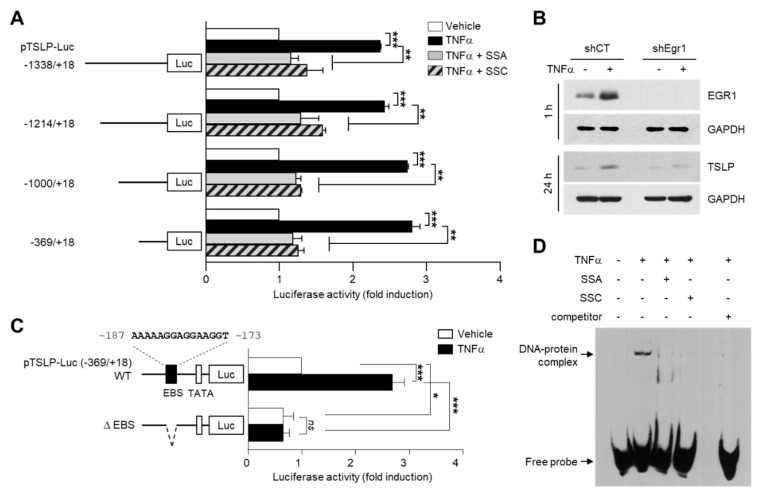
Role of EGR1 in TNF-α-induced *TSLP* transcription. (**A**) HaCaT cells were transfected with 0.2 μg of a series of 5′-deletion constructs of the *TSLP* promoter. After 24 h, the cells were treated with 10 ng/mL of TNF-α in the absence or presence of 1 μM SSA or 20 μM SSC for an additional 8 h, and luciferase promoter-reporter activities were measured. (**B**) Immunoblotting was performed to examine the effects of control scrambled shRNA (shCT) and EGR1-specific shRNA (shEgr1) on TNF-α-induced EGR1 and TSLP protein expression. (**C**) HaCaT cells were transfected with wild-type and EBS-deleted pTSLP-Luc (−369/+18) plasmid. After 24 h, the cells were stimulated with TNF-α (10 ng/mL) for an additional 8 h, and luciferase activities were analyzed. (**D**) HaCaT cells were treated with TNF-α (10 ng/mL) in the absence or presence of SSA (1 μM) or SSC (20 μM). After 1 h, nuclear extracts (3 μg) were incubated with a biotinylated EGR1-binding oligonucleotide probe (50 fmol) in the absence or presence of an unlabeled probe (competitor, 2500 fmol). Data are expressed as means ± SD (*n* = 3). * *p* < 0.1, ** *p* < 0.01, *** *p* < 0.001 by Sidak’s multiple comparisons test. WT, wild-type; ∆EBS, deletion of EGR1-binding sequence from −187 to −173.

TNF-α stimulates *TSLP* promoter activity [25,26]. To investigate whether SSA and SSC inhibit TNF-α-induced *TSLP* promoter activity, we used a series of 5′-deletion constructs of the *TSLP* promoter region, including −1338/+18, −1214/+18, −1000/+18, and −389/+18, wherein the *TSLP* promoter drives luciferase reporter activity [26]. In accordance with a previous report [26], transient transfection of these promoter-reporter constructs into HaCaT cells demonstrated a significant (all *p <* 0.001) increase in luciferase reporter activity upon TNF-α stimulation, which was abrogated by SSA or SSC treatment (Figure 4A). Notably, SSA and SSC inhibited the promoter-reporter activity in the shortest construct, −369/+18. These data suggest that the response element for SSA and SSC is located between −369 and +18 in the *TSLP* promoter region. 

Previously, we reported that the transcription factor EGR1 transactivates the *TSLP* promoter through the EGR1-binding sequence (EBS), located in the region between −206 and −187, in response to TNF-α stimulation [26]. In this study, we observed that EGR1 knockdown using shRNA (shEgr1) reduced TNF-α-induced TSLP expression (Figure 4B). To evaluate whether EBS is the functional element of SSA and SSC, we used a mutant construct in which EBS (∆EBS) was deleted from the −369/+18 construct. Deletion of EBS resulted in a significant reduction in basal and TNF-α-induced *TSLP* promoter activity compared to that in the wild-type (WT) construct (Figure 4C). Next, we determined whether SSA and SSC affected the binding of EGR1 to EBS at −369/+18 region by performing the electrophoretic mobility shift assay (EMSA). HaCaT cells were treated with TNF-α in the presence or absence of SSA or SSC, and nuclear extracts were incubated with a biotinylated EBS oligonucleotide probe. The DNA-binding proteins were analyzed using streptavidin-conjugated horseradish peroxidase. The cells were treated with unlabeled EBS competitors at a fifty-fold excess (2.5 pmol) concentration to indicate the specific reaction of the DNA–protein complex formation. We found that TNF-α-induced formation of the DNA–protein complex was substantially reduced upon SSA or SSC treatment (Figure 4D). These data demonstrate that EGR1 transactivates EBS at −206/−187 region of the *TSLP* promoter, suggesting that EBS is the functional response element of SSA and SSC involved in TNF-α-induced suppression of the *TSLP* promoter activity. 

### 2.4. SSA and SSC Inhibit TNF-α-Induced EGR1 Expression

**Figure 5:**
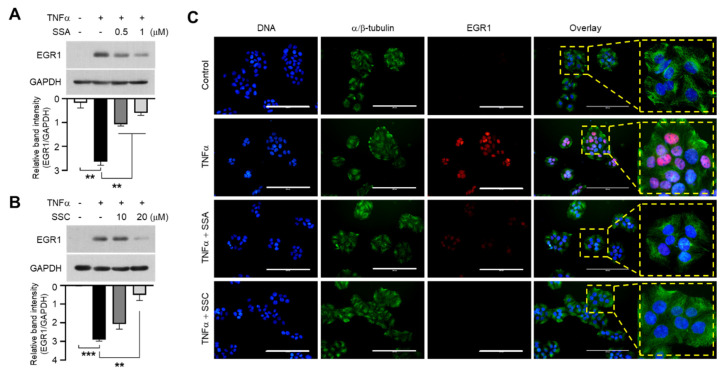
SSA and SSC decrease TNF-α-induced increase in EGR1 protein levels. (**A**,**B**) HaCaT cells were treated with 10 ng/mL of TNF-α for 1 h in the absence or presence of 0, 0.5, or 1 μM SSA (**A**) and 0, 10, or 20 μM SSC (**B**)**.** Protein levels of EGR1 were determined by immunoblotting using specific antibodies. GAPDH was used as the internal control. The band intensities of EGR1 were normalized relative to those of GAPDH using ImageJ software. The quantitation data are presented as means ± SD (*n* = 3). ** *p* < 0.01, *** *p* < 0.001 by Sidak’s multiple comparisons test. (**C**) HaCaT cells, cultured on coverslips, were treated with 10 ng/mL of TNF-α for 1 h in the absence or presence of 1 μM SSA or 20 μM SSC, and immunofluorescence staining was performed using anti-EGR1 (red) and anti-α/β-tubulin (green) antibodies. Hoechst 33258 (1 µg/mL) was used for counterstaining the nuclei (blue). Scale bars, 200 μm.

**Figure 6:**
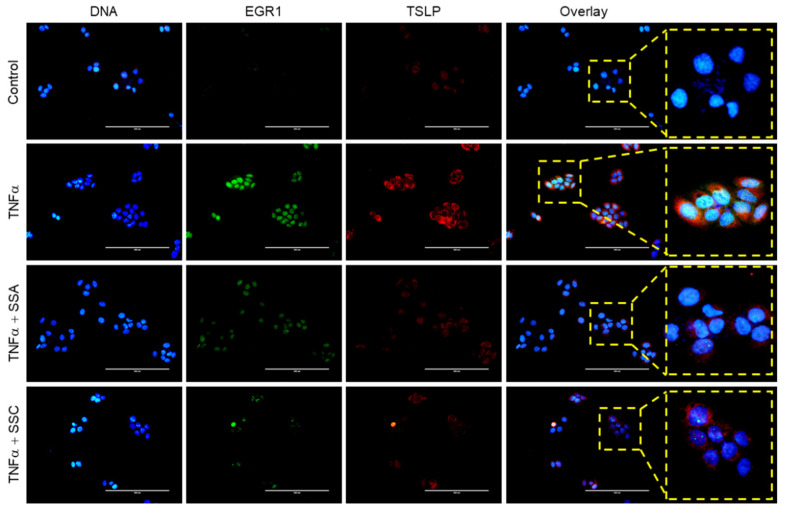
Subcellular localization of TNF-α-induced EGR1 and TSLP proteins. HaCaT cells were treated with 10 ng/mL of TNF-α for 12 h in the absence or presence of 1 μM SSA or 20 μM SSC. Immunofluorescence staining determined the localization of EGR1 (green) and TSLP (red). Nuclei were counterstained using 1 µg/mL Hoechst 33258 (blue). Scale bars, 200 μm.

To investigate whether SSA and SSC modulate EGR1 expression, we treated serum-starved HaCaT cells with TNF-α for 1 h in the presence or absence of SSA or SSC. We found that treatment with both SSA (Figure 5A) and SSC (Figure 5B) significantly (*p <* 0.01) suppressed TNF-α-induced EGR1 accumulation in a dose-dependent manner. Fluorescence immunocytochemical staining showed that TNF-α-induced nuclear localization of EGR1 was effectively reduced by SSA or SSC pretreatment (Figure 5C). In addition, SSA or SSC concomitantly decreased TNF-α-induced nuclear localization of EGR1 and cytoplasmic TSLP expression in the same cell, as observed by immunofluorescence staining (Figure 6). These data suggest that the downregulation of EGR1 expression is functionally linked to TSLP reduction in response to SSA or SSC pretreatment in HaCaT cells. 

### 2.5. SSA and SSC Abrogate TNF-α-Induced Activation of Mitogen-Activated Protein Kinase Signaling

**Figure 7:**
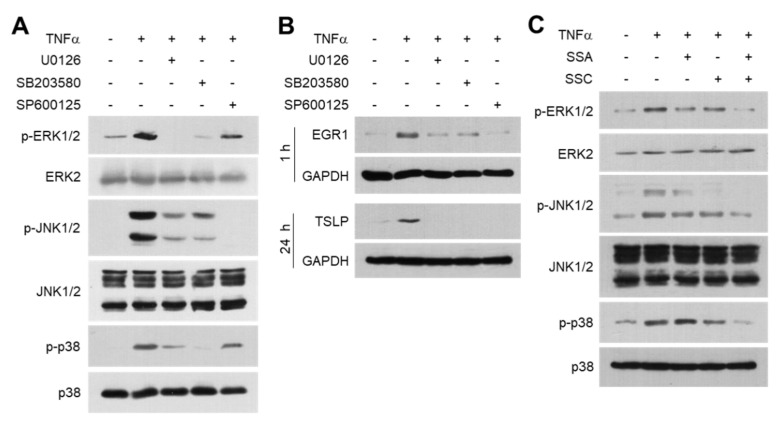
Effects of SSA and SSC on the inhibition of TNF-α-induced mitogen-activated protein kinases (MAPKs). HaCaT cells were pretreated with 10 μM of U0126, 20 μM of SB203580, or 25 μM of SP600125 for 30 min and then treated with 10 ng/mL of TNF-α. (**A**) After 10 min, whole-cell lysates were immunoblotted using phospho-specific and total MAPK antibodies. (**B**) The protein levels of EGR1 (at 1 h) and TSLP (at 24 h) were measured by immunoblotting. (**C**) HaCaT cells were treated with 10 ng/mL of TNF-α for 10 min in the presence or absence of 1 μM SSA or 20 μM SSC. Immunoblotting was performed using phospho-specific and total MAPK antibodies.

EGR1 expression is regulated by the mitogen-activated protein kinase (MAPK) pathway in various cell types [27,28,29]. As TNF-α stimulates the MAPK pathway in HaCaT cells [26], we examined whether SSA and SSC modulate MAPK signaling. In accordance with a previous report [26], we also observed that TNF-α-induced phosphorylation of ERK1/2, JNK, and p38 kinase was decreased upon pretreatment with U0126 (MAPK inhibitor), SB203580 (p38 kinase inhibitor), and SP600125 (JNK inhibitor) (Figure 7A). Notably, these inhibitors reduced TNF-α-induced EGR1 and TSLP expression (Figure 7B). Next, we determined whether SSA and SSC modulated MAPK activity. We found that SSA reduced TNF-α-induced phosphorylation of ERK1/2 and JNK1/2, while SSC inhibited that of all three MAPKs (Figure 7C). These data suggest that SSA and SSC abrogate TNF-α-induced EGR1 and TSLP expression by inhibiting MAPKs.

### 2.6. Topical Application of SSA or SSC Ameliorates Atopic Dermatitis-like Skin Inflammation in BALB/c Mice Challenged with 2,4-Dinitrochlorobenzene

**Figure 8:**
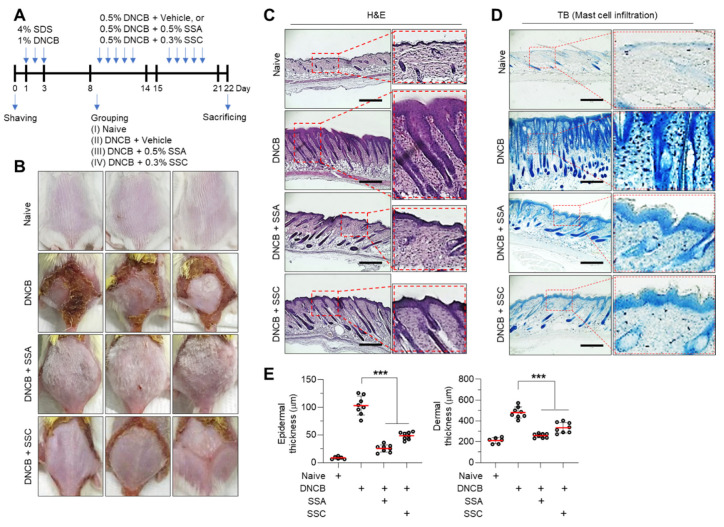
Effect of topical application of SSA and SSC on the amelioration of dinitrochlorobenzene (DNCB)-induced AD-like skin lesions in BALB/c mice. (**A**) Experimental design for the induction of AD-like skin lesions. (**B**) Representative images of the back skin of BALB/c mice, challenged with DNCB in the presence or absence of 0.5% SSA or 0.3% SSC, were acquired on day 22. (**C**,**D**) Paraffin-embedded skin tissues were prepared and stained with hematoxylin and eosin (**C**) and toluidine blue (**D**). Scale bars, 200 μm. (**E**) Epidermal and dermal thicknesses were measured using ImageJ software. Data are presented as means ± SD (Vehicle group, *n* = 6; Treated group, *n* = 8). *** *p* < 0.001 by Sidak’s multiple comparisons test.

We further evaluated the effect of topical application of SSA and SSC on the suppression of TSLP expression in vivo. As TSLP expression is upregulated in AD-like skin lesions in BALB/c mice challenged with DNCB [26], we used a mouse model of DNCB-induced skin inflammation. BALB/c mice were repeatedly sensitized with DNCB solution on the back skin (Figure 8A). DNCB caused AD-like inflammatory symptoms in this experimental model, which were reduced by repeated topical application of SSA or SSC (Figure 8B). Histological analysis by hematoxylin and eosin (H&E) staining demonstrated that application of SSA and SSC reduced DNCB-induced hyperkeratosis in the epidermis (Figure 8C). Morphometric analysis indicated that SSA and SSC significantly (*p* < 0.001 in all cases) suppressed DNCB-induced epidermal and dermal thickness (Figure 8D). The infiltration of inflammatory cells is dispensable for skin inflammation in an allergen-induced mouse model [30]. Marked infiltration of immune cells, such as Th lymphocytes and mast cells, was observed in DNCB-induced AD-like skin lesions [25]. To determine the effect of topical application of SSA and SSC on the infiltration of inflammatory cells, mast cells and neutrophils were stained with toluidine blue (TB). Consistent with a previous report, the number of TB-stained cells was increased in the dermis of DNCB-treated mice, which was substantially reduced by SSA or SSC treatment (Figure 8E). These data suggest that SSA and SSC ameliorate chronic inflammatory responses in AD-like skin lesions in DNCB-induced BALB/c mice.

### 2.7. Topical Application of SSA and SSC Inhibited EGR1 and TSLP Expression in DNCB-Induced AD-like Skin Lesions in BALB/c Mice

**Figure 9:**
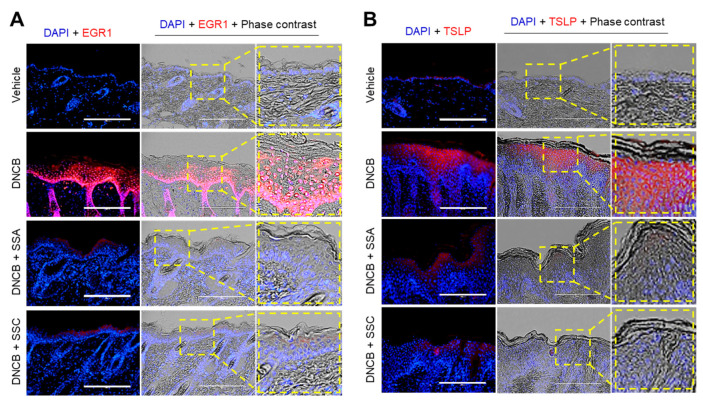
SSA and SSC reduce the enhanced expression of both EGR1 and TSLP in DNCB-challenged BALB/c mice. BALB/c mice were treated with vehicle (70% ethanol), DNCB + 0.5% SSA, or DNCB + 0.3% SSC. Paraffin-embedded skin tissue sections were deparaffinized and incubated with anti-EGR1 (**A**) and anti-TSLP primary antibodies (**B**), followed by incubation with rhodamine red-X-conjugated secondary antibody (red). Nuclei were counterstained with Hoechst 33258 (blue). Scale bars = 200 μm.

Finally, we determined the effect of topical application of SSA and SSC on suppressing EGR1-regulated TSLP expression in DNCB-induced AD-like skin lesions. Immunofluorescence microscopy showed that both EGR1 (Figure 9A) and TSLP (Figure 9B) staining intensities were markedly increased in the epidermal layer of DNCB-treated skin compared to those of the vehicle-treated group. Notably, topical application of SSA or SSC substantially reduced the staining intensities of both proteins compared to those of the vehicle-treated group. These data support the notion that SSA and SSC ameliorate skin inflammation by downregulating EGR1-regulated TSLP expression in AD-like skin lesions.

## 3. Discussion

AD is a chronic itchy, inflammatory dermatitis that affects both children and adults and remained extremely difficult to treat until recently. One of the most prominent clinical symptoms of AD is chronic robust itching behavior, which causes sleep deprivation and mental distress, resulting in a substantial reduction in the quality of life [31]. Peripheral sensory nerves branching from the dorsal root ganglia transmit itch perception to the central nervous system, triggering scratching behavior [32]. Various pruritogenic factors, including histamines, cytokines, neuropeptides, proteinases, and neurotransmitters, are associated with the pathophysiology of pruritic symptoms in patients with AD [33]. Of these, Th2 cytokines, such as IL-4, IL-13, and IL-31, directly stimulate sensory neurons by binding to their cognate receptors expressed on neurons that elicit the scratch behavior [34,35,36]. TSLP is highly expressed in the skin lesions of patients with AD. It induces Th2 cell differentiation and triggers the production of Th2-mediated cytokines, such as IL-4, IL-5, and IL-13, and activates various immune cells, leading to the development of AD [7,37]. Furthermore, TSLP directly activates sensory neurons and mediates itch sensitization [13]. Therefore, TSLP is considered a therapeutic target for the treatment of allergic diseases, including AD [7]. 

Traditional Chinese herbal medicine has been used in clinical practice for several years to treat AD [38]. However, the widespread use of traditional medicines for clinical applications is limited, as herbal medicines contain multiple bioactive ingredients with unknown components [39]. This study showed that SSA and SSC identified from *Radix Bupleuri* reduced TNF-α-induced TSLP expression at the transcriptional level by inhibiting MAPK signaling-mediated transcriptional activation of EGR1 in HaCaT keratinocytes. Interestingly, SSA was approximately 20-fold more effective than SSC in the inhibitory effect on TNF-α-induced TSLP mRNA expression (Figure 2A,B). Due to insufficient data comparing the mode of action of SSA and SSC, it is currently not possible to explain the different effectiveness between these two compounds. SSA and SSC share the same triterpenoid structures, but their side-chain groups are different. The sidechain of SSA contains the disaccharide of glucose-fucose, whereas SSC contains a trisaccharide of glucose–glucose-rhamnose [19]. Thus, one possible explanation is that different carbohydrate structures between SSA and SSC may affect the cell membrane permeability. Further in-depth studies are required to address this issue.

It has been reported that *TSLP* transcription is regulated by various transcription factors, including vitamin D3 receptor, NF-κB, AP1, and EGR1 [26,40,41,42]. Using a series of deletion constructs of the *TSLP* promoter region, we found that the cis-acting element responsible for the effects of SSA and SSC was located between the −369 and +18 region in the *TSLP* promoter. Previously, we demonstrated that the EBS located at −206/−187 bp is critical for TNF-α-induced *TSLP* transcription [26]. EGR1 is an inducible transcription factor that plays a crucial role in pathophysiological responses in a mouse model of DNCB-induced AD-like skin lesions [25]. Furthermore, EGR1 is known to regulate IL-17A-induced psoriasin expression [43] and IL-33-induced TSLP expression [44] in keratinocytes during skin inflammation. Therefore, we speculated that SSA and SSC might inhibit TNF-α-induced *TSLP* transcription via EGR1 inhibition. To test this hypothesis, we examined the role of EGR1 in TNF-α-induced TSLP expression. We confirmed that EGR1 knockdown abrogated TNF-α-induced TSLP expression (Figure 3B) and treatment with SSA or SSC abrogated TNF-α-induced EGR1 DNA-binding activity (Figure 3C). Our findings are concordant with a previous report demonstrating that chrysin-induced suppression of TSLP expression is associated with the downregulation of EGR1 expression in HaCaT keratinocytes [26]. 

It has been well established that ERK1/2, JNK1/2, and p38 kinase mediate mitogen- and TNFα-induced EGR1 expression in various cell types [45]. We also observed that inhibition of the three major MAPKs using pharmacological inhibitors effectively reduced TNF-α-induced EGR1 and TSLP expression (Figure 6). Moreover, SSA and SSC inhibited TNF-α-induced phosphorylation of ERK1/2, JNK1/2, and p38 kinase (Figure 6). In addition, we confirmed that the topical application of SSA or SSC reduced DNCB-induced AD-like skin lesions in BALB/c mice. Accordingly, our results suggest that SSA and SSC suppress TSLP expression through downregulation of MAPK-mediated EGR1 expression in HaCaT keratinocytes. Nevertheless, we cannot rule out the possibility that SSA and SSC may also act via MAPK independent mechanisms to block TSLP expression.

In summary, suppression of EGR1-regulated TSLP expression in keratinocytes might be attributable to the anti-inflammatory effects of SSA and SSC on AD-like skin lesions.

## 4. Materials and Methods

### 4.1. Materials

SSA and SSC were purchased from Sigma-Aldrich (St. Louis, MO, USA). TNF-α was obtained from ProSpec-Tany TechnoGene Ltd. (Ness Ziona, Israel). The firefly luciferase assay system was purchased from Promega (Madison, WI, USA). The web-based program MatInspector (http://www.genomatix.de/ (accessed on 17 December 2020)) was used for promoter analysis. Anti-TSLP antibody was obtained from Novus Biologicals (Centennial, CO, USA), and anti-EGR1, phospho-ERK1/2 (Thr202/Tyr204), phospho-p38 (Thr180/Tyr182), and phospho-JNK1/2 (Thr183/Tyr185) antibodies were obtained from Cell Signaling Technology (Danvers, MA, USA). The anti-GAPDH antibody was purchased from Santa Cruz Biotechnology (Dallas, TX, USA). Secondary antibodies conjugated to Alexa Fluor 488 and rhodamine red-X were obtained from Jackson ImmunoResearch Laboratories (West Grove, PA, USA). The MAPK, p38 kinase, and JNK inhibitors (U0126, SB203580, SP600125), DNCB, TB, and an H&E staining kit were purchased from Sigma-Aldrich.

### 4.2. Cells and Cell Culture

Immortalized human keratinocyte HaCaT cells were obtained from Cell Lines Service (Eppelheim, Germany) and cultured in Dulbecco’s modified Eagle’s medium supplemented with 10% fetal bovine serum (HyClone, Logan, UT, USA) and 0.5% penicillin-streptomycin (Sigma-Aldrich).

### 4.3. Cytotoxicity Assay 

Cell viability assay was performed using the CCK-8 (Dojindo Molecular Technologies, Gaithersburg, MD, USA) according to the manufacturer’s instructions. Briefly, HaCaT cells (3 × 10^3^ cells/sample) were treated with either the vehicle or different concentrations of SSA or SSC (0, 1, 5, 10, and 20 µM). After 24 h, CCK-8 solution was added, and the cells were incubated for an additional hour. The CCK-8 solution contains water-soluble tetrazolium salt WST-8 (2-(2-methoxy-4-nitrophenyl)-3-(4-nitrophenyl)-5-(2,4-disulfophenyl)-2H-tetrazolium, monosodium salt), which is colorless, but produces orange-colored WST-8 formazan dye when reduced by dehydrogenase in cells. The amount of WST-8 formazan dye was measured using the Emax Endpoint ELISA Microplate Reader (Molecular Devices, Sunnyvale, CA, USA) at 450 nm, which indicated the number of living cells.

### 4.4. RT-PCR and qPCR

Total RNA was extracted from HaCaT cells using the TRIzol RNA extraction kit (Invitrogen, Carlsbad, CA, USA). First-strand cDNA was synthesized using the iScript cDNA synthesis kit (Bio-Rad, Hercules, CA, USA). RT-PCR was performed using reverse transcriptase (Promega) and gene-specific PCR primers, as follows:

- TSLP forward, 5′-TAG CAA TCG GCC ACA TTG CCT-3′;
- TSLP reverse, 5′-GAA GCG ACG CCA CAA TCC TTG-3′;
- GAPDH forward, 5′-CCA AGG AGT AAG AAA CCC TGG AC-3′;
- GAPDH reverse, 5′-GGG CCG AGT TGG GAT AGG G-3′.

The thermal cycling conditions were as follows: 94 °C for 5 min, followed by 30 cycles of denaturation at 94 °C for 30 s, annealing at 58 °C for 30 s, and elongation at 72 °C for 1 min. The amplified PCR products were electrophoresed on a 2% agarose gel and visualized under a UV transilluminator.

qPCR was performed using the iCycler iQ system and IQ SYBR Green Supermix kit (Bio-Rad) and validated using commercial qPCR primers and SYBR Green-based fluorescent probes specific for TSLP (id: qHsaCIP0030468) and GAPDH (id: qHsaCEP0041396) purchased from Bio-Rad. The relative expression levels of *TSLP* mRNA were normalized to those of GAPDH using the software provided by the manufacturer (Bio-Rad).

### 4.5. Immunoblot Analysis

HaCaT cells were lysed using the RIPA buffer containing 50 mM of Tris-HCl (pH 7.4), 1% NP-40, 0.25% Na-deoxycholate, 500 mM of NaCl, 1 mM of EDTA, 1 mM of Na_3_VO_4_, 1 mM of NaF, 10 mg/mL of leupeptin, and 1 mM of PMSF. The protein extracts were electrophoresed on 8 or 12% sodium dodecyl sulfate (SDS) polyacrylamide gels and transferred onto nitrocellulose membranes. After incubation with appropriate primary and secondary antibodies, the enhanced chemiluminescence detection system (GE Healthcare, Piscataway, NJ, USA) was used to develop the blots. The intensities of the immunoreactive bands were quantified using ImageJ software, version 1.52a. The relative band intensities were calculated as a ratio to GAPDH band intensity.

### 4.6. Immunofluorescence Microscopy

HaCaT cells cultured on coverslips were treated with the drugs, fixed, permeabilized, and incubated with primary antibodies (for 24 h at 4 °C), followed by incubation with secondary antibodies (for 1 h at 25 °C) and 1 μg/mL Hoechst 33258 (for 1 h at 25 °C). The fluorescently stained cells were observed using the EVOS FL fluorescence microscope (Advanced Microscopy Group, Bothell, WA, USA).

### 4.7. Human TSLP Promoter-Reporter Constructs and Generation of Internal Deletion of EGR1-Binding Sequence (EBS) 

Construction of a series of deletion constructs of the human *TSLP* promoter region-spanning nucleotides −1338/+18, −1214/+18, −1017/+18, and −369/+18 has been described elsewhere [26]. Internal deletion of EBS from −187 to −173 bp (∆EBS) from the pTSLP-Luc(−369/+18) plasmid was performed using the inverse PCR method, which amplifies the entire plasmid DNA except for the region to be deleted, using two primers in inverted tail-to-tail directions [46]. The sequences of the two primers used to generate the ∆EBS construct were as follows:

−172F: 5′-GAGGGAAATTCCTGATGACT-3′;
−187R: 5′-GCTTTGCCCTAAACACTTC-3′.

The sequence of the deletion construct was verified by DNA sequencing (Macrogen, Seoul, Korea).

### 4.8. Luciferase Promoter-Reporter Assay

HaCaT cells were cultured in 12-well plates and transiently transfected with 0.2 μg *TSLP* promoter-reporter constructs using Lipofectamine 2000 (Invitrogen), according to the manufacturer’s instructions. After 24 h of transfection, the cells were pretreated with different concentrations of SSA or SSC for 30 min, followed by the addition of 10 ng/mL of TNF-α. After 8 h, firefly luciferase activity was measured using the Dual-Glo luciferase assay system (Promega) with the pRL-null plasmid encoding Renilla luciferase (Promega) according to the manufacturer’s instructions. Luciferase activity was measured using the dual luminometer (Centro LB960; Berthold Tech, Bad Wildbad, Germany). 

### 4.9. EGR1 Knockdown by RNA Interference 

HaCaT cells were transfected with lentiviral shRNA (TRCN_0000273850; MISSION^®^ shRNA; Sigma-Aldrich) targeting EGR1 (shEgr1), according to the manufacturer’s instructions. After two weeks, knockdown of EGR1 expression was verified by immunoblot analysis.

### 4.10. Electrophoretic Mobility Shift Assay (EMSA)

DNA binding activity of EGR1 was examined using the LightShift Chemiluminescent EMSA kit (Thermo Fisher Scientific, Waltham, MA, USA). A biotinylated deoxyoligonucleotide probe (5′-CAA AAA GGA GGA AGG TGA GGG AA-biotin-3′) corresponding to EBS was synthesized by Macrogen. Nuclear extracts were prepared from HaCaT cells using the Nuclear and Cytoplasmic Extraction kit (Thermo Fisher Scientific). Nuclear extracts (3 µg) were incubated with the biotin-EBS probe (50 fmol) and salmon sperm DNA (Sigma-Aldrich) in the absence or presence of unlabeled probes as a competitor (2500 fmol). DNA-protein complexes were electrophoresed on non-denaturing 6% polyacrylamide gels and visualized using the Amersham ECL western blotting detection kit (GE Healthcare Life Science, Chicago, IL, USA).

### 4.11. DNCB-Induced Atopic Dermatitis-like Skin Lesions in BALB/c Mice and Topical Application of SSA and SSC

Male BALB/c mice (7-week-old) were purchased from Orient Bio Inc. (Seongnam, Korea). The mice were divided into four groups: Group I, naive; Group II, DNCB + vehicle (phosphate-buffered saline (PBS)); Group III, DNCB + 0.5% SSA; Group Ⅳ, DNCB + 0.3% SSC. The mouse in vivo study was performed as described previously, with minor modifications [26]. Briefly, all mice except for Group I were sensitized with 4% SDS on the back skin to disrupt the skin barrier; after 4 h, the SDS-sensitized areas were treated with 1% DNCB dissolved in acetone:olive oil mixture (1:3, *v*/*v*). The mice were treated with DNCB once daily for 3 days on the SDS-sensitized area and then rested for 4 days. SSA and SSC were dissolved in 70% ethanol at a concentration of 0.5% and 0.3%, respectively. After a challenge with 0.5% DNCB, 120 µL of 0.5% SSA or 0.3% SSC was dropped (Group II and Group IV). After sacrificing all mice on day 22, paraffin-embedded tissue sections were prepared. All animal experiments were performed according to the protocols approved by the Konkuk University Institutional Animal Care and Use Committee (approval number KU19129).

### 4.12. Fluorescence Immunohistochemical Staining

Fluorescence immunohistochemical staining was performed as described previously with minor modifications [47]. Briefly, paraffin-embedded skin sections were deparaffinized with xylene, hydrated with graded ethanol, and incubated with 1 mM EDTA (pH 8.0) for 20 min at 60 °C, followed by blocking with 7% goat serum for 1 h. The sections were then probed with primary antibodies against TSLP (1:100 dilution) and EGR1 (1:100 dilution) overnight at 4 °C. After rinsing with PBS, the slides were incubated with rhodamine Red-X-conjugated secondary antibody (Jackson ImmunoResearch Laboratories, 1:300 dilution) for 1 h at 25 °C. After counterstaining the nuclear DNA with Hoechst 33258 solution, the slides were mounted with coverslips and fluorescence mounting medium (ProLong Gold Antifade Reagent; Invitrogen). Fluorescence images were captured using the EVOS FL fluorescence microscope (Advanced Microscopy Group).

### 4.13. Statistical Analysis

Data are presented as mean ± standard deviation (SD). Statistical analyses were performed using one-way analysis of variance (ANOVA) followed by Dunnett’s or Sidak’s multiple comparisons test using GraphPad Prism version 9.0.1 (GraphPad Software, Inc., La Jolla, CA, USA). The results with a *p* value less than 0.05 indicated statistically significant differences for all analyses.

## 5. Conclusions

Our study demonstrated that SSA and SSC identified from *Radix Bupleuri* suppressed TNF-α-induced TSLP expression at the transcriptional level by inhibiting MAPK signaling-mediated transcriptional activation of EGR1 in HaCaT keratinocytes. Topical application of SSA or SSC ameliorated AD-like skin lesions in a mouse model challenged with DNCB. In conclusion, SSA or SSC could be used as effective and promising candidates for topical therapeutic application in chronic skin inflammatory diseases such as AD. 

## Data Availability

Data available upon reasonable request.
